# Supplementary material for: Analysis of PPARγ Signaling Activity in Psoriasis
Source: Int J Mol Sci. 2021 Aug 10;22(16):8603. doi: 10.3390/ijms22168603 (PMC8395241; doi:10.3390/ijms22168603)
Supplement: Supplementary file 1 [file ijms-22-08603-s001.zip › Supplemental materials_Analysis of PPARg signaling activity in psoriasis/Pathway models/Models images and html files/PPARG expressed in cells assotiated with psoriasis/115827.html]

epithelium


# Tissue epithelium

|  |  |
| --- | --- |
| URN | urn:agi-ncimtissue:C0014609 |
| Total Entities | 27 |
| Connectivity | 14653 |
| Name | epithelium |

---

|  |  |
| --- | --- |
| ChildConcepts | respiratory epithelium |
|  | cervix epithelium |
|  | glandular epithelium |
|  | ductal epithelium |
|  | gastrointestinal epithelium |
|  | multilaminar epithelium |
|  | unilaminar epithelium |
|  | pseudostratified epithelium |
|  | kidney epithelium |
|  | urachal epithelium |
|  | outer enamel epithelium |
|  | odontogenic epithelium |
|  | neuroepithelium |
|  | urethra epithelium |
|  | squamous epithelium |
|  | pigment epithelium |
|  | sensory epithelium |
|  | ocular epithelium |
|  | uterine tube epithelium |
|  | anal epithelium |
|  | thyroid follicular epithelium |
|  | apical domain of the epithelium |
|  | epithelial plica |
|  | lateral domain of the epithelium |
|  | basal domain of the epithelium |
|  | ciliated epithelium |
|  | mesothelium |

---

|  |  |
| --- | --- |
| Pathway | PPARG expressed in cells assotiated with psoriasis |

---

|  |  |
| --- | --- |
| MedScan ID | 6802426 |

---

|  |  |
| --- | --- |
| Alias | epithelial |
|  | epithelias |
|  | textus epithelialis |
|  | epithelial linings |
|  | epithelial tissues |
|  | epithelium |
|  | epithelial lining |
|  | epithelia |
|  | epithelial tissue |
|  | epitheliums |

---

|  |  |
| --- | --- |
| NCIm ID | C0014609 |

---
